# Comparative Outcomes of Delayed i-PRF Combination with Nanofracture in the Treatment of Large Chondral Defects in the Knee

**DOI:** 10.3390/medicina61101849

**Published:** 2025-10-15

**Authors:** Özgür Başal, James G. Jefferies, Jure Serdar, Mahmut Nedim Doral

**Affiliations:** 1 Department of Orthopedics and Traumatology, Medical Park Gebze Hospital, 41700 Kocaeli, Türkiye; 2 Department of Orthopedics and Traumatology, Maidstone & Tunbridge Wells NHS Trust, London ME16 9QQ, UK; jamesgjefferies@gmail.com; 3 Department of Orthopedics and Traumatology, University Hospital Centre Zagreb, 10000 Zagreb, Croatia; jserdar00@gmail.com; 4 Department of Orthopedics and Traumatology, Hacettepe University, 06210 Ankara, Türkiye; mndoral@hacettepe.edu.tr

**Keywords:** bone marrow stimulation, cartilage regeneration, injectable PRF, MOCART 2.0, AMADEUS, chondral defect, nanofracture

## Abstract

*Background and Objectives*: This study aimed to evaluate whether delayed intra-articular application of injectable platelet-rich fibrin (i-PRF) improves clinical and radiologic outcomes compared to nanofracture alone. *Materials and Methods*: A total of 76 patients with ICRS Grade III–IV femoral condyle or trochlear cartilage defects larger than 2 cm^2^ were enrolled in this prospective controlled study. Patients were allocated into two groups: Group 1 (*n* = 40) underwent nanofracture followed by delayed intra-articular i-PRF injection at three weeks postoperatively, while Group 2 (*n* = 36) underwent nanofracture alone. Preoperative MRI was evaluated using the AMADEUS grading system. Clinical outcomes—including WOMAC and IKDC scores—were assessed at baseline and at 6, 12, and 24 months postoperatively. MOCART 2.0 scoring was used to evaluate cartilage repair at ≥12 months follow-up. *Results*: Both groups demonstrated significant functional improvement according to the IKDC and WOMAC scores. However, Group 1 showed a significantly greater improvement in WOMAC total score at final follow-up (Group 1: 20.1 ± 4.3 vs. control: 23.2 ± 3.4; *p* = 0.0008). No statistically significant differences were found between groups in IKDC score (*p* = 0.238), Tegner score (*p* = 0.776), or time to return to daily activities (*p* = 0.401). Baseline demographic, radiological, and intraoperative variables were comparable between groups (*p* > 0.05 for all). Radiologic outcomes based on the mean MOCART 2.0 scores were 57.1 and 50, respectively, in group 1 and group 2 (*p* = 0.0316). These results showed significantly improved results in group 1 according to the MRI evaluation. *Conclusions*: In patients with large chondral defects (>2 cm^2^), delayed intra-articular i-PRF injection following nanofracture may improve mid-term functional and radiological outcomes, particularly in pain and symptom relief. This regenerative strategy enhances cartilage repair potential during the early healing phase without adding surgical complexity.

## 1. Introduction

Chondral defects of the knee, especially those larger than 2 cm^2^, present a substantial treatment challenge because of their low intrinsic healing capacity [1]. Effective joint-preserving techniques are crucial because if left untreated, these lesions frequently develop into severe osteoarthritis [1,2].

Although bone marrow stimulation (BMS) methods like microfracture are still considered first-line treatments, their long-term results are not always consistent. Fibrocartilage lacks the biomechanical resilience of native hyaline cartilage, causing functional deterioration in >50% of patients by 10 years [3,4]. A more recent BMS technique called nanofracture makes deeper, narrower subchondral bone channels to improve the recruitment of progenitor cells such as mesenchymal stem cells (MSCs) and decrease subchondral bone edema [1]. The biological shortcomings of the healing microenvironment are not addressed by nanofracture alone, despite its technical benefits [1].

It has been shown that the chondrocytes and fibroblasts present in the knee environment can synthesize collagen under appropriate conditions and that the MSC potential revealed by bone marrow stimulation also contributes to the formation of new cartilage [5,6]. At this point, it is necessary to provide the appropriate environment and shift the microenvironment towards an anabolic direction.

Autologous platelet-rich fibrin (PRF) is a promising cartilage repair adjuvant [7]. In injectable PRF (i-PRF), the three-dimensional fibrin matrix is enriched in leukocytes and cytokines (TGF-β, VEGF, and PDGF), crucially eluting sustained growth factor release over 7–14 days in contrast to platelet-rich plasma (PRP) [5,6,7]. These characteristics encourage the production of extracellular matrix, chondrocyte proliferation, and anti-inflammatory regulation [8,9]. Importantly, the liquid formulation of PRF also known as injectable form makes it possible to deliver the product in a minimally invasive manner, which has a significant advantage over solid PRF membranes in arthroscopic settings [5,7,8,9,10].

Delaying postoperative administration may maximize the therapeutic window for i-PRF, even though early intraoperative treatment showed potential. The first inflammatory phase (days 1–7) after a nanofracture gives way to regeneration (days 7–28) [9,11,12,13]. A delayed injection of i-PRF during this phase of the reparative process may have the following effects: it may enhance matrix deposition, counteract catabolic cytokines, and enhance MSC homing and differentiation [9,13].

Despite the potential advantages of i-PRF, no clinical research has assessed delayed i-PRF augmentation after significant chondral defects are treated by nanofracture. The purpose of this study is to determine whether or not the combination of nanofracture and delayed i-PRF results in improved structural and functional outcomes when compared to nanofracture alone.

## 2. Materials and Methods

### 2.1. Study Design and Ethical Approval

This prospective, single-center, comparative cohort study was conducted between January 2023 to December 2024. The study was approved by the Istinye University-affiliated Medical Park Hospital’s local ethical committees (Nr. 2025-001). Written informed consent was provided by each participant. All surgical interventions were performed by a single senior orthopedic surgeon specialized in knee (O.B.). Each patient was personally called for an interview, and questionnaires were mailed to each patient. Patient demographic, surgical, and imaging data were obtained by reviewing medical records.

### 2.2. Patient Selection

Inclusion criteria consisted of symptomatic patients with unilateral femoral condyle or trochlear chondral defects classified as ICRS Grade III–IV larger than 2 cm^2^. Patients were included who had a failed conservative treatment of at least six months, were aged between 18 and 60 years old, and had a BMI of ≤35 kg/m^2^. All patients had no prior surgical treatment for cartilage defects in the affected knee. All chondral defects were confirmed by magnetic resonance imaging (MRI). Articular cartilage defects were rated using the AMADEUS (Area Measurement And DEpth and Underlying Structures) scoring method, which provides a standardized, MRI-based evaluation of cartilage lesions. The scoring was performed on 1.5-T MRI scans performed before surgery, utilizing T2-weighted and fat-suppressed proton density sequences in the coronal and sagittal planes. Table 1 summarizes the distribution of cases with AMADEUS grade III and IV among the groups.

All patients underwent hematologic testing prior to surgery. Patients with platelet counts below 120,000 and hemoglobin levels less than 10 g/dL were excluded from the study. All patients routinely underwent a standard two-view standing knee X-ray during the initial examination, and Kellgren–Lawrence (K-L) grade 3 and 4 cases were excluded from the study. Patients with diffuse osteoarthritis, defined as K-L grade greater than 2, inflammatory arthritis, prior cartilage surgery, ligamentous instability, malalignment, and the presence of kissing lesions were excluded from the study.

### 2.3. Randomization and Group Allocation

There were two groups in this prospective comparative study. Computer-generated block randomization was used to evenly distribute patients into groups in a 1:1 ratio. Patients were categorized into the following two groups based on the intervention and control received:

Group 1 (Intervention Group): Delayed i-PRF combined with Nanofracture (*n* = 40)

Group 2 (Control Group): Nanofracture alone (*n* = 36)

Outcome assessors and patients were blinded to group allocation to minimize assessment bias. There was a slight imbalance between the groups (Group 1: *n* = 40; Group 2: *n* = 36) due to post-randomization loss and minor allocation imbalance. However, this did not affect the integrity of the randomization process, and the study preserved its internal validity without introducing systematic bias.

### 2.4. Surgical Procedure

#### 2.4.1. Nanofracture (Both Groups)

All patients underwent an arthroscopic nanofracture procedure performed under spinal or general anesthesia. Under sterile conditions, a pneumatic tourniquet was placed in a supine position with a pressure of 250–300 mmHg. A ring curette or shaver was used to remove the unstable cartilage and the calcified cartilage layer to create a stable vertical margin around the defect. This procedure was performed after diagnostic arthroscopy verified the location and characteristics of the chondral lesion.

**Figure 1:**
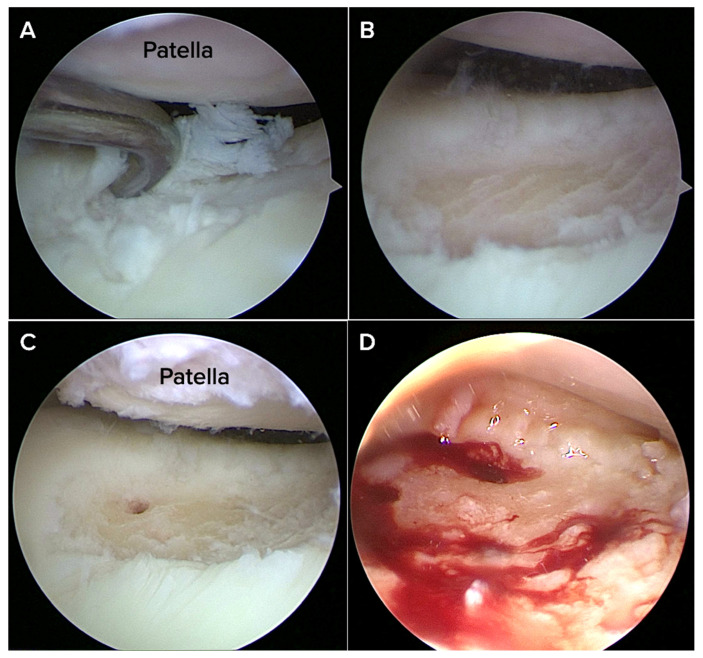
Arthroscopic images of a Grade IV chondral lesion located on the femoral trochlea of a 42-year-old male patient presenting with chronic anterior knee pain. A ring curette was used to debride unstable cartilage in order to create stable vertical margins. (**B**) The surface after the calcified cartilage layer has been removed. (**A**,**C**) nanofracture awl was used to induce controlled perforations that allowed access to the subchondral bone marrow. (**D**) Successful subchondral access and marrow stimulation are confirmed by the visible effusion of marrow elements from nanofracture sites.

A nano-fracture awl system (Nanofracture, Doratek^®^, Ankara, Turkiye) with a thin tip (about 1 mm) designed to minimize damage to the bone below the cartilage was then used to encourage healing in the bone underneath. The pre-calibrated awl was inserted perpendicularly into the defect base at approximately 5–10 mm depth, with perforations spaced 3–4 mm apart across the entire lesion bed (Figure 1).

Compared to conventional microfracture, this method allows for controlled marrow access with reduced bone compaction and thermal necrosis. Care was taken to avoid damage to adjacent cartilage. Hemostasis was achieved arthroscopically, and the joint was thoroughly irrigated before closure.

#### 2.4.2. i-PRF Preparation (Group 1)

In Group 1, all patients were instructed to discontinue the use of anti-inflammatory medications at least three days prior to the i-PRF injection. At the third week after surgery, a total of 20 mL of autologous venous blood was drawn under sterile conditions to prepare the injectable platelet-rich fibrin (i-PRF). The blood was distributed into sterile, plastic tubes without anticoagulant and centrifuged at a low-speed protocol (700 RPM (60× *g*) for 3 min) using an angled rotor system (35° rotor angle, 90 mm radius; Elektro-mag M815B, Istanbul, Turkey), following a previously described method [7].

A 7–8 mL of i-PRF was injected into the operated knee joint via the anterolateral soft spot using a 21G needle under sterile conditions. Based on wound-healing nature, the i-PRF injection was scheduled for the third postoperative week. Fibroblast infiltration and proliferation mainly start between days 3–7 and peak around weeks 1–3, which corresponds to the shift from acute inflammation to the early reparative phase in soft tissue and cartilage [14,15,16]. The i-PRF infiltration during this window utilizes the increased cellularity, angiogenesis, and extracellular matrix production-mediated by growth factors such as TGF-β and FGF-2-without increasing intra-articular pressure.

### 2.5. Postoperative Rehabilitation Protocol

A systematic rehabilitation procedure supervised by professional physiotherapists was followed by all patients.

Weeks 0–2: Patients were not permitted to weight-bear on the operated limb and continuous passive motion (CPM) therapy started postoperatively from 0 to 45° flexion to prevent knee joint stiffness, encourage blood flow and nourishment of cartilage.

Weeks 2–4: Patients progressed to partial weight-bearing with the introduction of active and assisted range of motion (ROM) exercises.

From Week 4, full weight-bearing was allowed and a quadriceps and hamstring strength training program began.

At follow-up appointments, adherence to the rehabilitation protocol was tracked, and all patients adhered to the same postoperative treatment plans.

### 2.6. Outcome Measurements

The clinical and imaging assessments were performed preoperatively, as well as at 6, 12, and 24 months after surgery.

#### 2.6.1. Primary Outcomes

The minimum clinically important difference (MCID) was defined as approximately 12 points for the subjective knee score estimated by the International Knee Documentation Committee (IKDC) and ≥1.5 points for the pain subscale of the Western Ontario and McMaster Universities Osteoarthritis Index (WOMAC), according to previously established thresholds [17,18]. The Tegner Activity Scale, which extends from 0 (no exercise because of knee issues) to 10 (high-level competitive sports engagement), was used to measure the preoperative activity level.

#### 2.6.2. Secondary Outcomes

The time taken to return to the pre-injury activity level or sports was recorded in months. Joint effusion, stiffness, and reoperation rate after surgery were also reported.

#### 2.6.3. Radiological Outcome Measures

Prior to cartilage repair, the AMADEUS (Area Measurement and Depth and Underlying Structures) score was used by a trained and blinded radiology examiner to assess preoperative 1.5-T MRI images acquired at our institution in order to measure the severity of chondral and osteochondral defects [19].

The International Cartilage Repair Society (ICRS) grading system was used for intraoperative grading [20]. The MOCART 2.0 (magnetic resonance observation of cartilage repair tissue, 0 = worst, 85 = best) score was used to evaluate the condition of hyaline cartilage or repaired tissue in all patients 12 months after surgery [21]. No cartilage mapping or additional radiological analysis was performed for this study. All MOCART measurements were carried out by an experienced musculoskeletal radiologist who works at the same institution but did not participate in the study design, patient selection, surgical procedures, or clinical evaluations.

### 2.7. Statistical Analysis

The required sample size was determined to be 33 patients in each group (66 patients overall), based on a two-sided *t*-test with an alpha level of 0.05, statistical power of 80%, and an expected effect size (Cohen’s d) of 0.7 derived from previously published studies on PRF and cartilage repair.

Independent *t*-tests were used to investigate continuous data, and chi-square (χ^2^) tests were used to evaluate categorical variables. Repeated-measures analysis of variance (ANOVA) was used to assess longitudinal data. MOCART 2.0 scores were examined using Welch’s independent samples *t*-test, which takes into consideration unequal variances between groups, in order to assess the quality of cartilage repair between the two groups. SPSS version 28 was used for all statistical analyses (IBM, Armonk, NY, USA). The threshold for statistical significance was *p* < 0.05.

## 3. Results

### 3.1. Clinical Outcomes

The study included 40 subjects in Group 1 and 36 subjects in Group 2. The mean preoperative International Knee Documentation Committee (IKDC) scores were 55 ± 5.6 in group 1 and 52 ± 6.3 in group 2, respectively. The mean preoperative WOMAC score was 54 ± 6.7 in Group 1 and 55 ± 4.2 in Group 2 (*p* = 0.434). Statistical analysis showed no significant difference between the groups for either the preoperative IKDC or WOMAC scores (*p* values > 0.05) (Table 2).

**Figure 2:**
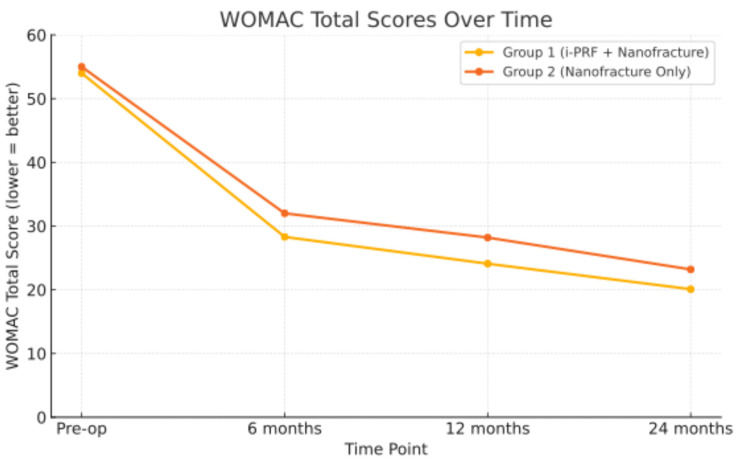
The WOMAC total scores for both treatment groups pre- and postoperative at 6th 12th and 24th months. Although both groups showed improvement after surgery, Group 1 (i-PRF + nanofracture) consistently had lower (better) WOMAC scores, particularly at 12 and 24 months.

At final follow-up, both groups showed clinical improvement in pain and functional outcomes. The IKDC scores for Group 1 (86.1 ± 4.8) and Group 2 (84.7 ± 5.4; *p* = 0.238) were not significantly different. There were also no statistically significant differences in WOMAC subscale scores for pain (3.45 ± 2.15 vs. 3.50 ± 2.1; *p* = 0.919), stiffness (1.4 ± 0.5 vs. 1.4 ± 1.5; *p* = 1.000), or difficulty (14.7 ± 3.7 vs. 15.0 ± 4.0; *p* = 0.736). However, a statistically significant improvement was observed in the WOMAC total score, favoring Group 1 (20.1 ± 4.3 vs. 23.2 ± 3.4, *p* = 0.0008), indicating better overall functional outcomes. The individual WOMAC subscales (pain, stiffness, and function) showed no statistically significant differences between groups; the total WOMAC score was significantly better in Group 1. Although the difference in total WOMAC score was statistically significant (*p* < 0.05), it did not exceed the MCID threshold for clinical relevance (Figure 2). This result is statistically valid and may be due to the fact that the total score is made up of a lot of data points from different areas, especially the function subscale, which has 17 items and has a bigger effect on the total score.

### 3.2. Baseline Characteristics and Patient Demographics

A total of 76 patients were involved in the study: 36 patients were in Group 2 (nanofracture only) and 40 patients were in Group 1 (delayed i-PRF plus nanofracture). Baseline demographics, such as age (45 ± 6.5 vs. 46 ± 3.8 years; *p* = 0.411), gender distribution (16M/24F vs. 10M/26F; *p* = 0.379), and body mass index (30 ± 5.05 vs. 31 ± 3.2 kg/m^2^; *p* = 0.301), did not significantly differ across groups. There was no statistical significance between the groups in any of the baseline demographic, clinical, or intraoperative variables (*p* > 0.05 in all comparisons), indicating that the groups were well-matched. The groups’ preoperative symptom durations were comparable (20.5 ± 4.8 vs. 19.2 ± 6.2 months; *p* = 0.314). Additionally comparable between groups were the ICRS classification, cartilage thickness at lesion margins, AMADEUS grade distribution (Grade 3/4), and chondral defect etiology (traumatic, degenerative, or unknown) (*p* > 0.05 for all). There was no significant difference in the distribution of defect locations (trochlear, medial femoral condyle, and lateral femoral condyle) (*p* = 0.998). The length of the surgery was comparable for both groups (48 ± 12.3 vs. 50.4 ± 10.6 min; *p* = 0.364). The distribution of related meniscal diseases and concurrent ACL injuries was equal (*p* > 0.05) (Table 3).

Group 1 and Group 2 had mean preoperative Tegner Activity Levels of 3.6 ± 1.3 and 3.5 ± 1.7 (*p* = 0.776), respectively. These mean levels of physical activity were comparable and were in line with light to moderate daily activities like walking or light labor. Group 1 and Group 2 did not differ substantially in the mean time for returning to daily activities (2.4 ± 1.6 months vs. 2.8 ± 2.4 months; *p* = 0.401).

### 3.3. Radiologic Outcomes

After a minimum of one year of follow-up, 47 (61.8%) of the 76 patients underwent an unscheduled MRI based on clinical indications. These MRIs were performed at the discretion of the operating surgeon in response to symptoms such as persistent effusion, stiffness, localized joint pain, mechanical symptoms (e.g., catching or locking), or reduced range of motion. MRI scans taken with a follow-up period of less than one year were not included in the MOCART 2.0 evaluation. Among those, 28 patients (59.6%) underwent MRI due to the presence of symptoms. The mean follow-up period was 15.3 months at the time of MOCART 2.0 analysis, and group 1’s and group 2’s (control) average MOCART 2.0 scores were 57.1 and 50, respectively. In comparison to the control group, Group 1 (delayed i-PRF) demonstrated significantly higher MOCART 2.0 scores, indicating higher-quality cartilage repair (*p* = 0.0238).

### 3.4. Return to Activity

The time to return to daily activities was comparable between treatments. On average, patients resumed normal activities at 2.4 ± 1.6 months in Group 1 and 2.8 ± 2.4 months in Group 2 (*p* = 0.401). This slight difference was not statistically significant. Both cohorts therefore returned to routine function in approximately 2–3 months, indicating that adjunct i-PRF did not accelerate or delay recovery time on a clinically meaningful level.

### 3.5. Complications

Among the 40 patients in Group 1, two had joint stiffness, while four presented with joint effusion. In Group 2, three of the 36 patients had joint effusion, while two presented with joint stiffness. No significant differences in the incidence of joint effusion or temporary stiffness were seen, and neither group experienced any severe postoperative complication. No patient was reoperated on due to these postoperative complications. Extended physical therapy was conducted to address joint stiffness, and repeated joint punctures were performed to alleviate joint effusion.

## 4. Discussion

To the best of our knowledge, this is the first study to evaluate delayed i-PRF administration in combination with nanofracture for chondral defects larger than 2 cm^2^. The timing of i-PRF infiltration seems to be important and may positively influence the quantity and quality of newly formed cartilage.

The early results of this study indicate that the combination of delayed i-PRF infiltration following three weeks of arthroscopic nanofracture procedure did not lead to superior outcomes compared to nanofracture alone for treating large cartilage injuries. However, this combination was associated with improved pain and function scores. This prospective study showed that both groups were able to demonstrate improvements in patients clinical scores at 2 years of follow-up.

The cytokines and growth factors contained in i-PRF, which are released gradually over time, create an appropriate microenvironment for cartilage repair. Applying i-PRF in the early reparative phase may help create new hyaline cartilage by encouraging fibroblasts to produce an adequate amount of collagen. i-PRF may have some benefits and drawbacks when compared to bone marrow stimulation methods such as microfracture, Autologous Chondrocyte Implantation (ACI), MCI, and AMIC. However, the existing literature still lacks adequate data. The beneficial effects of orthobiologic agents applied during surgery may not always be obtainable during the acute phase of trauma [22]. Delays in application are therefore very significant in this situation. Unlike treatments such as ACI, MCI, or AMIC commonly used for large cartilage lesions, i-PRF stands out as a minimally invasive and cost-effective treatment.

There are two methods to produce PRF: soluble and membrane. Although membrane PRF (M-PRF) is already employed as a biological scaffold, there are technical difficulties when using it in an arthroscopy setting. Conversely, the soluble form of PRF has a similar mode of action and can target cartilage defects, activate local fibrocytes and chondrocytes, and initiate regeneration within the microenvironment through the slow release of growth factors.

**Figure 3:**
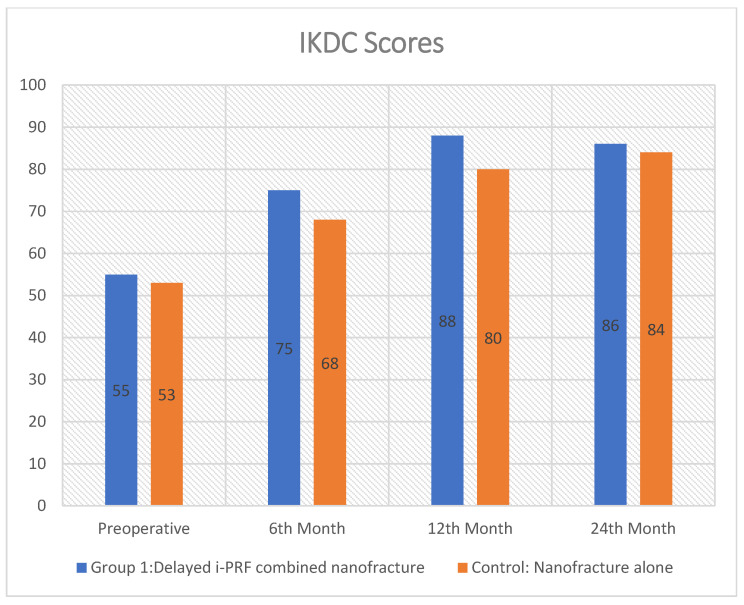
The IKDC scores obtained preoperatively and at 1, 6, 12, and 24 months are shown in the graphical abstract.

Patient-reported outcomes were evaluated using IKDC, WOMAC, and activity scores to assess clinical and functional metrics across distinct groups (Figure 3). Group 1 demonstrated statistically superior functional outcomes as measured by WOMAC, with the difference being clinically significant. The achieved improvement in WOMAC score, while statistically significant, did not exceed the minimum clinically important difference (MCID). This finding suggests that the benefit, although measurable, may not translate into a significant functional improvement for patients. Both treatments resulted in clinically significant improvements when compared to baseline preoperative outcomes.

Based on the thickness of the healthy cartilage margins, cartilage lesions were categorized as either shouldered or unshouldered in this study. Shouldered lesions were defined as having more than 3 mm of intact cartilage around the defect. Shouldered cartilage lesions, consisting of a greater amount of healthy cartilage at the defect margins, may provide an optimal environment for cartilage regeneration. The presence of these reliable margins potentially offers better structural support and promotes the integration of repair tissue, which could enhance the quality and durability of cartilage healing. This morphological contrast is important when assessing treatment outcomes and may influence the regenerative potential following interventions such as nanofracture combined with delayed i-PRF injection.

Intra-articular effusion is common during the acute trauma period after surgery. Fibroblast activity rises in the joint environment when the effusion goes down and the proliferative phase starts. It might be possible to promote the formation of more mature hyaline or hyaline-like cartilage by administering i-PRF during this period. The proliferative phase typically reaches its peak between weeks 2 and 4, with fibroblasts emerging as the predominant cell type by week 7. Unlike PRP, which provides a quick, transient boost, i-PRF is differentiated by an ongoing release of growth factors over several of weeks [11,14,15,23]. This prolonged release corresponds effectively with the proliferative healing phase, making the three-week mark an ideal time to utilize its regenerative potential.

This study has several limitations that should be acknowledged. First, the control group did not receive a placebo intra-articular injection such as saline or hyaluronic acid, which may have affected comparative interpretations. The MRI-based MOCART 2.0 scoring was limited to a small number of patients and primarily symptomatic individuals. This introduces a potential selection bias, as asymptomatic patients may have been underrepresented in the MRI-assessed subgroup. However, it is important to note that the distribution of symptomatic patients who underwent MRI was similar in both groups. Therefore, the MRI findings appear to be consistent with the overall clinical outcomes. Histological examination of the restored cartilage tissue was not included in the study, which restricts the capacity to evaluate the quality of newly formed cartilage directly. There were no second-look arthroscopic evaluations to see and directly examine the cartilage repair, which could have yielded more conclusive data about tissue integration and quality. The follow-up period of up to 24 months may be insufficient to fully understand the long-term reliability and clinical outcomes of the treatment, especially considering the long-term nature of cartilage repair.

## 5. Conclusions

This prospective comparative study indicates that delayed intra-articular injection of i-PRF at three weeks post-nanofracture significantly enhanced radiological outcomes in patients with large chondral defects (>2 cm^2^), as demonstrated by superior MOCART 2.0 scores (57.1 vs. 50.0; *p* = 0.0238) in comparison to nanofracture alone. Functionally, both groups exhibited significant improvement from baseline; however, the i-PRF group attained statistically superior WOMAC total scores (20.1 vs. 23.2; *p* = 0.0008). Nevertheless, this disparity did not attain the threshold for the minimal clinically important difference, hence constraining its clinical significance. No significant differences between groups were noted in the primary outcome measure (IKDC scores, *p* = 0.238), Tegner activity levels (*p* = 0.776), or time to return to daily activities (*p* = 0.401).

The adjunctive use of i-PRF was shown to be safe, with no severe complications or reoperations in either group. While these results indicate potential benefits for structural cartilage repair during the early healing phase, the absence of clinical superiority in functional outcomes warrants careful interpretation. Future placebo-controlled trials with long-term follow-up and histological evaluation are essential to conclusively determine the beneficial effects of delayed i-PRF augmentation in cartilage regeneration protocols.

## Figures and Tables

**Table 1:** Distribution of cases according to baseline characteristics and patient demographics.

	Group 1 (Delayed i-PRF Combined with Nanofracture)	Group 2 (Control) (Nanofracture Only)	*p* Values (*p* < 0.05)
Gender: male/female (*n*)	16/24	10/26	0.379
Age (mean, SD)	45 ± 6.5	46 ± 3.8	0.411
BMI (kg/m^2^)	30 ± 5.05	31 ± 3.2	0.364
Duration of Symptoms (months)	20.5 ± 4.8	19.2 ± 6.2	0.314
Etiology (*n*)			0.948
Traumatic	10	8	
Degenerative	22	20	
Unknown	8	8	
Occupation/Activity level (Pre-op Tegner Activity Level)	3.6 ± 1.3	3.5 ± 1.7	0.776
AMADEUS Grade (*n*)			0.655
Grade 3	28	33	
Grade 4	12	13	

**Table 2:** Clinical outcomes based on IKDC, WOMAC, and activity scores obtained at 24-month follow-up; * *p* < 0.05 is considered statistically significant.

Outcome Measure	Group 1 (Mean ± SD)	Group 2 (Mean ± SD)	*p* Value
IKDC Score	86.1 ± 4.8	84.7 ± 5.4	0.238
WOMAC Total Score	20.1 ± 4.3	23.2 ± 3.4	0.0008 *
WOMAC Pain	3.45 ± 2.15	3.50 ± 2.1	0.919
WOMAC Stiffness	1.4 ± 0.5	1.4 ± 1.5	ns
WOMAC Difficulty	14.7 ± 3.7	15.0 ± 4.0	0.736
MOCART 2.0	57.1 ± 8.9	50 ± 11.6	0.0238 *
Return to Daily Activities (months)	2.4 ± 1.6	2.8 ± 2.4	0.401
Complication n/rate	6 (15%)	5 (13.8%)	ns
Reoperation Rate	0	0	ns

**Table 3:** Surgical diagnostics and intraoperative measurements; * *p* < 0.05 is considered statistically significant.

	Group 1 (Intervention) N = 40	Group 2 (Control) N = 36	*p* Values *p* < 0.05 *
Defect Location (*n*)			0.998
Trochlear	20	18	
Medial femoral condyle	12	11	
Lateral femoral condyle	8	7	
Lesion Size, SD (cm^2^)	3.9 ± 1.2	3.8 ± 4.9	ns
Cartilage thickness at intact margins (*n*)			ns
≥3 mm (considered as shouldered)	12	11	
<3 mm (unshouldered)	28	25	
ICRS Classification (*n*)			0.889
Grade 3	25	24	
Grade 4	15	12	
Concomitant Procedures (*n*)			ns
ACL reconstruction	6	5	
Meniscal repair	26	20	
Partial meniscectomy	16	18	
Plicae resection	30	28	
Surgery Time (min)	48 ± 12.3	50.4 ± 10.6	0.364

According to the Outerbridge classification, “ns” = not significant.

## Data Availability

All data are contained within the article.

## Abbreviations

ACL	Anterior Cruciate Ligament
AMADEUS	Area Measurement And DEpth and Underlying Structures
BMI	Body Mass Index
BMS	Bone Marrow Stimulation
CPM	Continuous Passive Motion
FGF-2	Fibroblast Growth Factor 2
IKDC	International Knee Documentation Committee
ICRS	International Cartilage Repair Society
i-PRF	Injectable Platelet-Rich Fibrin
K-L	Kellgren–Lawrence
MCID	Minimum Clinically Important Difference
MOCART 2.0	Magnetic Resonance Observation of Cartilage Repair Tissue
MSCs	Mesenchymal Stem Cells
MRI	Magnetic Resonance Imaging
ns	Not significant
PDGF	Platelet-Derived Growth Factor
PRF	Platelet-Rich Fibrin
PRP	Platelet-Rich Plasma
ROM	Range of Motion
TGF-β	Transforming Growth Factor Beta
VEGF	Vascular Endothelial Growth Factor
WOMAC	Western Ontario and McMaster Universities Osteoarthritis Index
